# Determination of Bioactive Compounds, Antioxidant Capacity, Safety Assessment, and Antimicrobial Effect of *Tristerix corymbosus* Extracts

**DOI:** 10.3390/molecules30234610

**Published:** 2025-11-30

**Authors:** Katia Fernández Moreno, Gabriela Maturana, Sofía Blanco-Haros, Ulises Norambuena-Jopia, Gabriela Valenzuela-Barra, María Carolina Zúñiga-López, Jessica Bravo Garrido

**Affiliations:** 1 Laboratory of Bioactive Natural Products, School of Medicine, Center for Biomedical Research, University Diego Portales (UDP), Ejército 141, Santiago 8370007, Chile; katia.fernandez@udp.cl (K.F.M.); gamaturana@uchile.cl (G.M.); 2 Faculty of Health and Dentistry, Universidad Diego Portales, Ejército 278, Santiago 8340263, Chile; 3 Department of Inorganic and Analytical Chemistry, Faculty of Chemical and Pharmaceutical Sciences, University of Chile, Santiago 8380494, Chile; sofiablancoharos@gmail.com (S.B.-H.); mczuniga@ciq.uchile.cl (M.C.Z.-L.); 4 Department of Pharmacological Chemistry and Toxicology, Faculty of Chemical and Pharmaceutical Sciences, University of Chile, Santiago 8380000, Chile; ulises.norambuena@ug.uchile.cl (U.N.-J.); gabriela.m.valenzuela@ciq.uchile.cl (G.V.-B.)

**Keywords:** *Tristerix corymbosus*, extract toxicity, methanolic extract, flavonoids

## Abstract

Quitral (*Tristerix corymbosus*), a Chilean and Argentine parasitic mistletoe, is traditionally used by Mapuche natives to treat stomach ulcers, nervous disorders, and cholesterol reduction, although scientific support is scarce. Methanolic and chloroform extracts from its leaves and stems were prepared. Chemical analysis included antioxidant capacity assays (ORAC-FL and DPPH) and chromatographic determinations. The antimicrobial activity was tested against nine bacteria and two yeast strains. Additionally, cytotoxicity (hemolysis) and toxicity (against *Caenorhabditis elegans*) assays were performed. The results revealed that the methanolic leaf extracts had the highest ORAC-FL value, with DPPH assays showing solvent-dependent differences. Thirty-one compounds were tentatively identified, of which 61% were phenolic compounds, primarily flavonoids like quercetin and its derivatives. Antimicrobial results showed activity against Gram-positive bacteria (*Staphylococcus aureus*, *Listeria monocytogenes*, *Bacillus cereus*, and *Enterococcus faecalis*), but not against yeast *Candida guillermondii* and *Candida tropicalis*. Methanolic extracts induced dose-dependent erythrocyte hemolysis, while chloroform extracts showed no relevant cytotoxicity. Toxicity against *Caenorhabditis elegans* was also dose-dependent for methanolic extracts; leaf extract reduced survival at 50 mg mL^−1^ after 24 h. These findings partially validate some traditional uses, highlight the importance of solvent polarity in extraction and biological effects, and establish quitral as a flavonoid source.

## 1. Introduction

*Tristerix corymbosus*, commonly known as Quitral or Quintral, is a hemiparasitic plant of the Loranthaceae family, native to Chile, where it grows from the Atacama Region to the Los Lagos Region, including Juan Fernández Island [1]. It is typically found in the country’s temperate forests. The stem and leaves of this plant have been traditionally used as medicine by the Mapuche people in Chile, with effects including anti-inflammatory, wound-healing, hemostatic, blood lipid-lowering, and sedative properties [2,3]. Nevertheless, the traditional uses do not guarantee the safety of consuming the various conventional preparations, and conducting scientific research to evaluate their toxicity is essential. In this regard, and to the best of our knowledge, the toxicity of ethanolic extracts from *Tristerix longebracteatus* has been studied against *Artemia salina* only to date, with the results showing a LC50 value of 350.70 μg mL^−1^ [4].

Scientific work on this plant has primarily focused on its ecological importance in temperate forest ecosystems [5,6,7,8], resulting in limited information about its chemical composition and antimicrobial effects. In 2016, Simigriotis et al. [3] studied the phenolic profile and reducing power of flower and leaf extracts of the same *Tristerix* species from two locations, identifying 36 phenolic compounds, including several caffeoyl acids, procyanidins, and flavonols. Later, in 2019, Torres et al. [9] studied the difference in the presence of cardiac glycosides, tannins, flavonoids, steroids, quinones, saponins, triterpenes, the total phenolic content (TPC), the total flavonoid content (TFC), and the reducing power depending on the tree species parasitized by quitral. The antiproliferative activity of these flower and leaf extracts has also been tested on breast, prostate, and gastric cancer lines [10]. Very recently, Velásquez et al. determined the phenolic composition, antioxidant capacity (AC), and antibacterial effects on *Staphylococcus aureus* ATCC 25923, *Escherichia coli* ATCC 25922, and *Salmonella typhi* ATCC 700623 of the insoluble phenolic acid, free phenolic acids, and esterified phenolic fractions of the flowers of quitral [11]. The authors reported the presence and concentration of five phenolic acids and 5 flavonoids quantified by UPLC, as well as the inhibitory effect of the extracts on the growth of the three strains tested. The free and esterified extracts had significantly greater antimicrobial activity than the insoluble phenolic acid extracts, presumably because of the lower concentration of phenolic compounds.

So far, in all the previously cited scientific work on quitral, phenolic compounds in the different extracts tested are accountable for the biological activities. Phenolic compounds have been reported to have antioxidant, anti-inflammatory, antimicrobial, anticancer, and blood glucose regulation activity [12,13,14,15,16,17,18,19], among others. Hence, the use of these compounds in health-related uses has been an interesting topic for investigation in the last decade.

This work aimed to assess the AC, total content of flavonoids and phenolic compounds, bioactive compound identification by HPLC and UPLC analysis, antimicrobial activity, cytotoxicity, and toxicity of the methanolic and chloroform extracts of the leaves and stem of *Tristerix corymbosus*.

## 2. Results

### 2.1. Extraction

Extraction was carried out by maceration at room temperature for 24 h with methanol and chloroform separately, obtaining 6 extracts in total: QLM (Quitral leaf methanol), QLC (Quitral leaf chloroform), QSM (Quitral stem methanol) and QSC (Quitral stem chloroform). Table 1 resumes the extraction yield.

### 2.2. Antioxidant Potential

**Figure 1:**
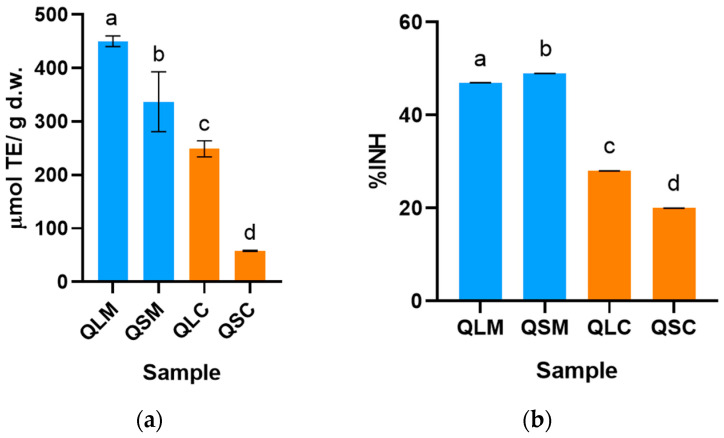
AC determined by (**a**) ORAC-FL and (**b**) DPPH methodologies in Tristerix corymbosus methanol and chloroform extracts from leaf and stem. All data is expressed as mean ± standard deviation (*n* = 3). QLM (Quitral leaf methanol), QLC (Quitral leaf chloroform), QSM (Quitral stem methanol), QSC (Quitral stem chloroform); %INH: Inhibition percentage. d.w.: dry weight; Different letters refer to statistically significant differences in datasets, *p* < 0.05.

As described previously, the antioxidant capacity (AC) was evaluated using the ORAC-FL and DPPH methodologies. The ORAC-FL results (Figure 1a) for the QLM extract show the highest AC of 450 ± 10 μmol TE g d.w.^−1^, statistically higher than every other sample. The sample with the lowest AC was QSC with 58 ± 1 μmol TE g^−1^ d.w. The methanolic extracts had higher AC than the corresponding chloroform extract.

As for the AC determined using the DPPH method and presented as inhibition percentage (%INH) of the radical (Figure 1b), all ANOVA comparisons were statistically significant (*p* < 0.05). The AC for the methanolic extracts is higher than that of the chloroform extract. Again, the QLM extract had the highest AC of all samples, with an AC of 55.00 ± 0.04% INH, while the lowest AC was found for QSC with 20.00 ± 0.01% INH of the DPPH radical.

### 2.3. Total Phenolic and Flavonoid Content

**Figure 2:**
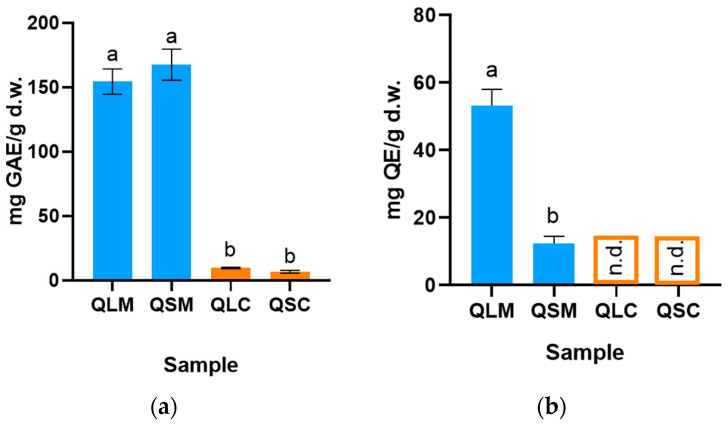
(**a**) Total phenolic content (TPC) and (**b**): flavonoid content (TFC) in Tristerix corymbosus methanol and chloroform extracts from leaf and stem. All data are expressed as mean ± standard deviation (n = 3). QLM (Quitral leaf methanol), QLC (Quitral leaf chloroform), QSM (Quitral stem methanol), QSC (Quitral stem chloroform); GAE: gallic acid equivalent; QE: quercetin equivalent; d.w.: dry weight; n.d.: not detected. Different letters refer to statistically significant differences in datasets, *p* < 0.05.

Figure 2 summarizes the TPC and TFC results for all extracts. As the instrumental response for chloroform extracts fell below the TFC calibration curve’s quantification limit, the concentration was augmented; however, the signal remained undetectable. Regarding the TPC, all methanolic extracts exhibited significantly higher TPC values than the respective chloroform extract. No statistically significant difference in TPC was observed among the methanolic extracts themselves. Conversely, the QLM extract showed significantly higher TFC compared to QSM.

### 2.4. Chemical Analysis

**Figure 3:**
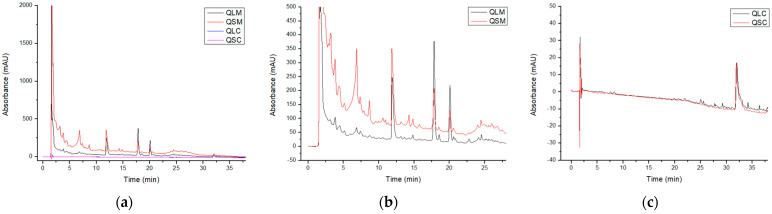
(**a**) Complete chromatograms for all studied extracts, (**b**) chromatograms for the chloroform extracts, and (**c**) chromatograms for the methanolic extracts. All chromatograms were registered at 278 nm.

Figure 3a summarizes the chromatograms registered at 278 nm for all extracts. The methanolic extracts exhibited a higher number of chromatographic peaks than the chloroform extracts, indicating that the chemical composition of the extracts comprises a greater proportion of polar molecules than apolar molecules. Most of the peaks in the methanolic extracts (Figure 3b) had retention times under 20 min; among them, most of the more intense peaks did not have spectra corresponding to phenolic compounds [20] except for the peaks at 17.3 and 20.0 min, which were identified as flavonoids by their UV spectra along with the less intense peaks at 9.2, 13.1, 13.6, 15.7, 18.5, 20.6, and 22.8 min [20]. Comparing the peaks with the same retention time, the methanolic extracts differ mostly in the intensity of the peaks rather than their identity, as they show the same UV-vis spectra. Some differences include the intensity of the peak at 20 min is higher in the QLM than in the QSM spectra. No relevant peaks were found in the chromatograms for the chloroform extracts (Figure 3c).

The HPLC-DAD chromatograms were compared to an in-house polyphenol standard mix as stated in the methodology. None could be identified, probably because of the technique’s limit of detection. The spectral analysis of the HPLC-DAD chromatograms suggests that at least 10 peaks can be identified as flavonoids, which is in concordance with the UPLC-MS tentatively identified compounds (Table 2). A total of 31 compounds were tentatively identified between both extracts: 26 phenolic compounds (22 flavonoids, 3 phenolic acids, 1 curcumin derivative), and 6 non-phenolic compounds. Among the flavonoids, 11 compounds were identified as methylated and/or glycosylated quercetin derivatives (compounds **7**, **9**, **12**, **14**, **17**, **18**, **21**, **22**, **26**, **27**, and **30**). Hyperoside, Isorhamnetin-3-O-galactoside, Ramnazin, and 3-methyl quercetin are among the most intense compounds found. Hyperoside has the highest intensity in both samples, while Isorhamnetin-3-O-galactoside, Rhamnazin, and 3-methylquercetin have the highest intensity in one of the two samples. Most of the compounds were identified in both samples, varying in signal intensity, except for compound 31 Avobenzone, which was found only in the QSM extract.

### 2.5. Biological Analysis

#### 2.5.1. Antimicrobial Activity

Table 3 summarizes the antimicrobial activity of the extracts as determined by the agar diffusion method. For Gram-negative bacteria, only low levels of inhibition were observed among the tested strains (ranging from 7.0 to 17.5 mm). In contrast, Gram-positive bacteria exhibited higher susceptibility to the extracts, while no antifungal activity was detected against the yeast strains. The QLM extract showed the strongest antibacterial effect, particularly against *L. monocytogenes* ATCC BAA-751. This extract demonstrated the highest activity among all samples tested, reaching nearly half of the inhibition observed for the reference commercial antibacterial compound (positive control), and displayed activity against four of the seven bacterial strains evaluated. *B. cereus* was identified as the most sensitive strain, with its growth inhibited by three of the four extracts to varying degrees.

Furthermore, Table 4 presents the Minimum Inhibitory Concentration (MIC) results obtained for the extracts selected based on their inhibition in the agar diffusion assay. Among these, the QLM and QSM extracts exhibited the lowest MIC values against *S. boydii*, highlighting their higher antibacterial potency.

#### 2.5.2. Cytotoxicity

**Figure 4:**
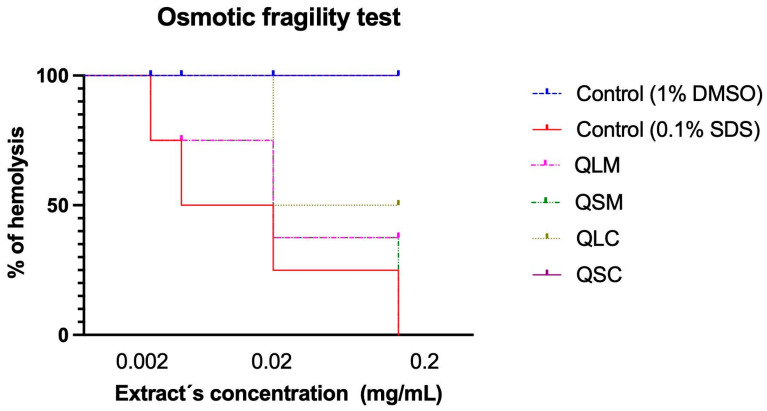
Osmotic fragility test of quitral extracts on human erythrocytes.

Cytotoxicity of quitral methanolic and chloroform extracts was tested in erythrocytes. Figure 4 summarizes their effect on the plasmatic membrane at different concentrations (without dilution, 1:10, 1:100, and 1:1000). This experiment measures the liberation of hemoglobin due to damage to the plasmatic membrane of erythrocytes. The absorbance measured at 530 nm was used to calculate the extract’s cytotoxicity. The methanolic extracts of leaf and stem (QLM and QSM) show greater cytotoxicity on erythrocytes than the chloroform extracts (QLC and QSC), which is dose-dependent: the higher the concentration, the greater the toxicity. In contrast, the chloroform extracts of leaf and stem (QLC and QSC) do not exhibit cytotoxicity on erythrocytes.

#### 2.5.3. Toxicity

**Figure 5:**
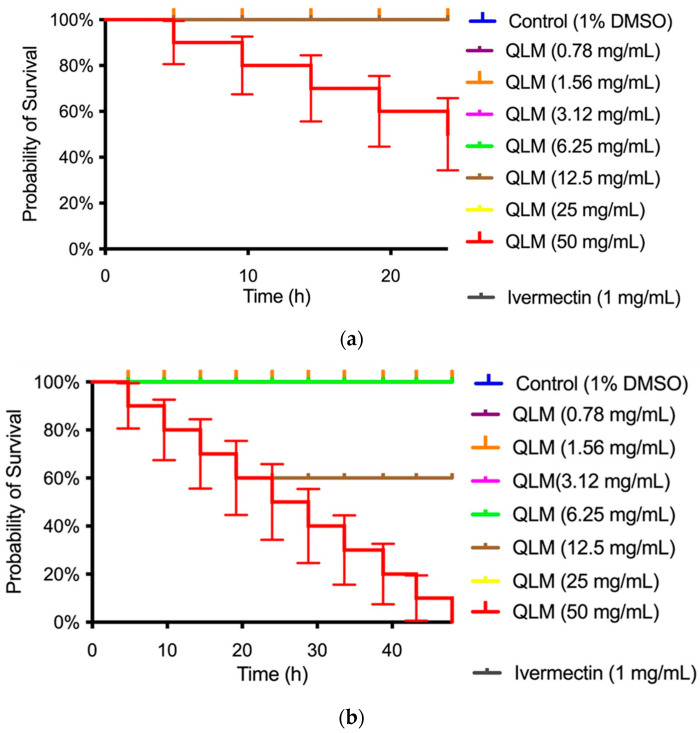
Percent survival of *Caenorhabditis elegans* exposed to QLM extract for (**a**) 24 h and (**b**) 48 h.

**Figure 6:**
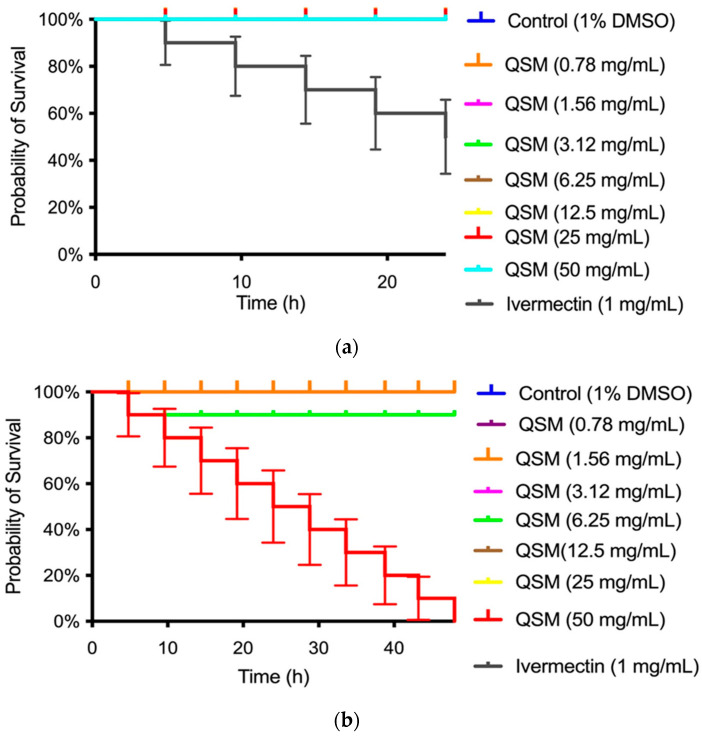
Percent survival of *Caenorhabditis elegans* exposed to QSM extract (**a**) for 24 h and (**b**) 48 h.

The observed toxicity of the QLM and QSM extracts against *C. elegans* was dose-dependent: the QLM extract at 24 h showed a decrease in survival at the concentration of 50 mg/mL (Figure 5a), while the QSM extract showed no toxicity at any concentration compared to the Ivermectin control (see Figure 6a).

## 3. Discussion

### 3.1. Antioxidant Capacity

The AC of a compound refers to its intrinsic capability to decelerate or impede the oxidation of a substrate, a process typically instigated by free radicals or reactive oxygen species (ROS). Polyphenols are an extensive family of naturally occurring and ubiquitous organic molecules that meet these conditions. Flavonoids, a subclass of polyphenols, have been widely studied in recent decades due to their health-beneficial properties, including antioxidant, anti-inflammatory, anticancer, hepatoprotective, and antimicrobial effects, among others [12,21,22,23,24,25,26,27]. Due to their chemical structure, flavonoids have poor solubility in nonpolar solvents, which is why the AC of the chloroform extracts (QSC and QLC) is lower than the AC for the methanolic extracts (QSM and QLM), as shown in Figure 1. This also correlates with the significantly higher TPC and TFC of the methanolic extracts compared to the chloroform extracts (Figure 2).

In this work, the QLM extract showed the highest AC in the ORAC-FL assay (450 ± 10 μmol TE g^−1^ d.w.), and the QSM extract showed the highest AC in the DPPH assay (20.00 ± 0.01%). At first glance, this does not correlate with the significantly higher TPC values (highest for QSM extract) (Figure 2a) but does correlate with the TFC values. It is essential to note that AC assays measure the total AC of the extracts, which typically implies the contribution of non-phenolic antioxidants, including vitamins, amino acids, and low-molecular-weight acids such as citric acid (Table 1). The fact that the QLM extract has the higher ORAC-FL and TFC values may suggest a higher contribution to the AC from the flavonoid portion than from other antioxidant compounds, but to establish the veracity of this idea, further analysis is required.

As expected, the TPC for the methanolic extracts was significantly higher than the chloroform extracts. The TFC for the chloroform extracts was not detected despite the increase in concentration performed (Figure 2b). TPC showed no significant difference between QSM and QLM, with the former having significantly higher TFC. Simirgiotis et al. report a TPC of 37.3 ± 0.9 mg GAE g^−1^ d.w. and 25 ± 1mg GAE g^−1^ d.w. for the leaf and flower acidified methanolic extracts of flowers and leaves of *Tristerix tetrandus extracts*, this is approximately seven times lower than the values reported in this work (Figure 2) for the QLM extract [3]. Regarding the TFC values, Simirgiotis et al. reported 27 ± 3 mg QE g^−1^ d.w. and 18 ± 2 mg QE g^−1^ d.w. for the leaf and flower extracts, respectively. This data are more similar to the data shown in Figure 2 for the QLM extract (12 ± 2.4mg QE g^−1^ d.w). The difference in sample collection year, location, and extraction method may account for these differences, as the chemical profile also varies [3]. It is also known that the Folin–Ciocalteu method for TPC quantification is susceptible to interference by any reducing compound able to reduce the Folin reagent, including reducing sugars, acids, and vitamins, among others [28].

Studies in other plant species have shown a similar tendency as in the quitral methanolic extracts analyzed in the present work. In another study performed by Atawodi et al. 2010, the authors compared the AC and phenolic composition of methanolic extracts from leaves, roots, and stems, obtained by maceration, from *Moringa oleifera* Lam [29]. The results showed a higher AC for the leaves than the stems [29]. This tendency correlates with the ORAC-FL values shown in Figure 1a for both methanolic and chloroform extracts. On the contrary, DPPH results (Figure 1b) follow a different trend, which can be accounted for by the difference between the AC assays. On one hand, in the ORAC assays, the predominant AC mechanism is Hydrogen Atom Transfer, which is carried out in a phosphate-buffered water solution at pH 7.4, and the AC is measured against oxygen-centered radicals (ROO^●^ and RO^●^), which tries to mimic physiological conditions [30]. On the other hand, in the DPPH inhibition assay, the mechanisms involving electron transfer are more predominant; the reaction medium is methanol, and the AC is tested against the artificial nitrogen-centered radical, DPPH. Hence, the AC information given by different AC assays may differ but should be considered complementary.

### 3.2. Chemical Composition

The chemical composition of any natural product relates directly to its properties and biological effects. As shown in Figure 3, the chemical composition of the QLM and QSM extracts differs in the number and intensity of their respective chromatographic peaks, the most notable being the compound at 6.4 min, whose concentration is higher in the QSM extract than in the QLM extract. Also, the QSM extract shows a higher number of peaks at retention times lower than 10 min, meaning a higher number of polar compounds in comparison with QLM. Other differences include the less intense peaks at 7.8 min, 9.2 min, 10.5 min, and 15.7 min, among others. The difference in chemical composition obeys the biological function of every plant organ, for example, Brozdowski et al. were able to identify 14 cinnamic acids, 2 flavonones, 12 flavanols, and 21 flavonols in the leaf methanolic extract of *Prunus serotina* Ehrh against the 10 cinnamic acids, 1 flavanone, 6 flavanols, and 24 flavonols in the flower methanolic extract [31]. Simirgiotis et al. were able to identify 6 anthocyanidins in flower extract of *Tristerix tetrandus* and 30 non-anthocyanidin phenolic compounds in the leaves extract, although it appears the authors only searched for anthocyanidins in the flower extract [3]. On the contrary, the similarities between samples may be connected to the common synthetic pathways between plant organs, and include the peaks at 12 min, 18 min, and 20 min with varying intensities.

Table 2 summarizes the major compounds found in the QSM and the QLM extracts. A total of 31 compounds were tentatively identified, 26 of which correspond to phenolic compounds. 22 compounds were identified as flavonoids, corresponding to 71% of the identified compounds. 12 compounds, 39% of the total identified compounds, were quercetin-related, and 8 (26%) kaempferol-related (Table 2). Flavonoids are a type of phenolic compound ubiquitous in the plant kingdom that are synthesized by plants by the shikimate pathway and the phenylacetate pathway. These compounds play important roles in plant homeostasis, serving as signaling molecules and defense against biotic and abiotic agents [32,33,34]. Quercetin is widely known for its AC and health-promoting properties [35], and is commonly found as glycosides in plants, which improves its bioavailability [36]. In recent years, quercetin has been studied for its applications in medicine [37] the food industry, and technology. Some interesting new investigations include the synthesis of quercetin derivatives for its use in medicine, including metal quelation, acylation, and nanoparticles, among others [38]. Some interesting new investigations include the synthesis of quercetin derivatives, including metal quelation, acylation, and nanoparticles, among others, for its use in medicine [38]. The glycosylation and methylations in quercetin modify its biological effects and efficiencies significantly [39]. For example, it is well stablished that one major contributor to the AC of quercetin is the 3′,4′-dihidroxy substitution in the B ring; if one of these hydroxyl groups were to be methylated, the AC would drop [40], which is the case for Isorhamnetin (compounds 14 and 21). Upon oral intake of flavonoids, glycosylation prevents flavonoid absorption in the small intestine, but if the glycoside is glucose, absorption occurs through the glucose transporter [36].

### 3.3. Biological Activity

The antimicrobial activity of the quitral methanolic extracts (QLM and QSM) showed a clear differential effect depending on the type of microorganism. As shown in Table 3, Gram-positive bacteria were more susceptible to the extracts than Gram-negative bacteria and yeasts. Regarding the MIC values evaluated against selected bacteria based on the inhibition observed in the agar diffusion assays, the QLM and QSM extracts exhibited a greater ability to inhibit *S. boydii* (Table 4). This finding is consistent with previous reports indicating that Gram-negative bacteria often exhibit higher resistance due to their outer membrane, which acts as a permeability barrier to many antimicrobial compounds [41,42]. The lower susceptibility of yeasts may also be attributed to differences in cell wall composition and efflux pump mechanisms [43].

Among all extracts, the QLM extract exhibited the strongest antibacterial effect, especially against *Listeria monocytogenes* ATCC BAA751 (Table 3). This result is noteworthy, as *L*. *monocytogenes* is a pathogenic bacterium associated with foodborne illness, and its susceptibility to plant-based antimicrobials has gained increasing interest [44]. The fact that QLM inhibited the growth of four out of seven bacterial strains tested highlights its broad-spectrum potential, suggesting that leaf-derived compounds may possess higher concentrations or more active combinations of antimicrobial phytochemicals.

Interestingly, *Bacillus cereus* was the most sensitive strain, responding to five of the six extracts. This aligns with findings from other studies where essential oils and methanolic extracts derived from medicinal plants exhibited significant inhibition of *B. cereus*, possibly due to the bacterium’s relatively permeable cell wall and lack of robust defense mechanisms [45,46].

The lack of antifungal activity observed in yeast strains suggests that the bioactive compounds present in the extracts may not effectively target the ergosterol synthesis pathway or disrupt fungal membrane integrity, mechanisms commonly exploited by antifungal agents [47]. Further studies should explore alternative extraction solvents or synergistic combinations to enhance antifungal properties.

Overall, these results support the antimicrobial potential of methanolic plant extracts, particularly leaf extracts, against Gram-positive bacteria, reinforcing their application as natural preservatives or therapeutic agents, especially considering the rise of antibiotic resistance.

The cytotoxicity assay using erythrocytes as a model system revealed clear differences in the hemolytic activity between the methanolic and chloroform extracts of quitral. As shown in Figure 4, the methanolic extracts of leaf (QLM) and stem (QSM) caused a dose-dependent hemolytic effect, with higher concentrations resulting in greater disruption of the erythrocyte membrane, as indicated by increased hemoglobin release. In contrast, the chloroform extracts (QLC and QSC) exhibited no measurable cytotoxicity at any tested concentration.

Erythrocyte hemolysis is a well-established indicator of membrane integrity and is commonly used to assess the biocompatibility and potential cytotoxicity of natural compounds [48,49]. The membrane-disrupting effects observed with the methanolic extracts suggest the presence of polar phytochemicals capable of integrating or destabilizing the lipid bilayer, leading to hemolysis [49]. These compounds may include flavonoids, saponins, and phenolic acids, which are often extracted efficiently with polar solvents such as methanol [50].

On the other hand, the absence of hemolytic activity in the chloroform extracts indicates that the less polar compounds present in these fractions are not able to compromise erythrocyte membrane integrity. This difference underscores the importance of solvent polarity in extracting bioactive compounds with specific biological effects [51]. While chloroform may extract lipophilic compounds with antimicrobial or antioxidant properties, its extracts appear to be biocompatible with erythrocytes, suggesting a safer profile for systemic applications.

These results are consistent with prior studies showing that methanolic plant extracts can exhibit stronger cytotoxic or hemolytic effects compared to non-polar extracts [52,53]. Therefore, the potential therapeutic use of quitral methanolic extracts must be carefully evaluated in terms of selectivity and safety, particularly when considering systemic administration routes.

*C. elegans* is a valuable in vivo model for assessing compound toxicity due to its conserved biological pathways with mammals [54]. In this regard, QLM and QSM extracts exhibited dose-dependent toxicity in *C. elegans*. Specifically, the QLM reduced survival at 50 mg mL^−1^ after 24 h (Figure 5). The QSM extract, in contrast, showed no observable toxicity across all tested concentrations when compared to the Ivermectin control (Figure 6). *C. elegans* has also been extensively used to evaluate the biosafety of nanoparticles, offering insight into sublethal impacts on development, intestinal and neuronal function, immune responses, and reproduction [55]. These findings reinforce the utility of *C. elegans* as a robust whole-animal model for toxicological studies, providing a cost-efficient and ethically favorable alternative to mammalian testing. Consistent results have been documented in other investigations involving essential oils from Chilean plants, which exhibited toxicity only at higher doses (25–50 mg mL^−1^), confirming their generally low toxic potential [56,57,58].

The major compounds identified (hyperoside, isorhamnetin-3-O-galactoside, rhamnazin, and 3-methylquercetin) exhibit a complementary biological profile that supports the antioxidant, antimicrobial, hemolytic, cytotoxicity, and overall toxicity activities observed in the extract.

In this work, several quercetin derivatives have been tentatively identified in QLM and QSM (Table 2). Among the more intense MS signals, hyperoside (quercetin-3-O-galactoside) has demonstrated strong AC and cytoprotective effects in vitro by enhancing HO-1 expression and the endogenous antioxidant system in H_2_O_2_-induced oxidative stress models, particularly in pulmonary and other cell types. It has also shown antimicrobial effects against both Gram-positive and Gram-negative bacteria, likely through membrane disruption mechanisms. Isorhamnetin-3-O-galactoside, a methylated derivative of quercetin, has been reported to activate the Nrf2/HO-1 pathway in hepatocytes, enhancing glutathione levels and phase II detoxifying enzymes, thereby protecting against oxidative stress-induced damage. These properties are consistent with the low hemolytic toxicity observed in the red blood cell model used in this work. Another quercetin derivative, Rhamnazin (3′,7-dimethylquercetin), although less frequently studied in isolation, is structurally related to isorhamnetin and has demonstrated antioxidant and antimicrobial properties in plant-derived extracts. Its presence likely contributes synergistically to the biological activity observed in the extracts. 3-Methylquercetin, the aglycone of isorhamnetin, also displays potent free radical scavenging activity, metal chelation, apoptosis induction in tumor cells, and minimal cytotoxicity in non-tumoral models. Its potential to inhibit key bacterial enzymes further supports its antimicrobial activity.

In the present study, the absence or low degree of red blood cell cytotoxicity aligns with the presence of these compounds, which are known for their cytoprotective properties and low intrinsic toxicity. The extract’s overall antioxidant activity is likely enhanced by the synergistic effects of hyperoside, isorhamnetin, and rhamnazin, all of which are capable of activating pathways such as Nrf2-HO-1, boosting detoxifying enzymes, and neutralizing reactive oxygen species. Similarly, the antimicrobial effects observed may be attributed to the ability of hyperoside and methylated flavonoids to interfere with essential bacterial functions [59,60].

Although general toxicity remained low in our assays, dose dependence must be considered. For instance, high sustained doses of hyperoside have been associated with renal effects in animal studies. Therefore, future cytotoxicity assays at higher concentrations are recommended to establish safety margins for therapeutic applications.

## 4. Materials and Methods

### 4.1. Sample Collection, Identification and Extraction

In January 2022, leaves and stems of Quitral (*Tristerix corymbosus* (L.) Kuijt, Loranthaceae family) were collected from La Fuente, Santa Cruz, O’Higgins (34°22′19″ S 71°07′28″ O). The botanist Pamela Ramírez identified the species, leaving an herbal testimony at the Playa Ancha University, Botany laboratory, Valaparaíso, Chile (VALPL_PLV 2383).

The samples were air-dried at room temperature to a constant weight, then each sample was powdered using a mortar and stored in a dark location. Extraction was carried out by maceration at room temperature for 24 h with methanol and chloroform separately, obtaining 6 extracts in total: QLM (Quitral leaf methanol), QLC (Quitral leaf chloroform), QSM (Quitral stem methanol), and QSC (Quitral stem chloroform). All extracts were dried and stored in the dark until use.

### 4.2. Antioxidant Potential Analysis

#### 4.2.1. ORAC-FL

The extracts’ AC was evaluated using the ORAC-FL method proposed by Ou et al. [61] with some modifications. Fluorescein was used as the fluorescent probe. Each extract was diluted with phosphate buffer (pH 7.4) to an instrumentally appropriate concentration. Then, 25 μL of each diluted extract was mixed with 150 μL of fluorescein in phosphate buffer (40 nM) in a 96-well white polystyrene microplate. Blanks were prepared as samples, but with a 25 μL phosphate buffer instead of the sample. The microplate was placed in a Synergy HT multi-detection microplate reader (Bio-TekInstruments, Winooski, VT, USA) and then incubated for 7 min at 37 °C. The radical reaction was initiated upon adding 25 μL of an 18 mM solution of AAPH in phosphate buffer, using a multichannel pipette. Then, fluorescence in each well was recorded every minute for 240 min, after gentle shaking, by measuring the emissions from the top of the microplate at an excitation wavelength of 485 nm and an emission wavelength of 520 nm. The time series of fluorescence decay was integrated, and the resulting area under the curve (AUC) was normalized by subtracting the AUC obtained in the blank experiment. After plotting, the sample AUC was compared with that obtained for Trolox, and the results were expressed as μmol Trolox equivalent per gram of dry weight (μmol TE g^−1^ d.w.).

#### 4.2.2. DPPH Radical Scavenging Activity

The DPPH scavenging capacity was spectrophotometrically monitored as described elsewhere with some modifications [62]. Briefly, 10 μL of each extract was added to 190 μL of a DPPH solution in methanol (40 mg L^−1^) directly in each well of a 96-well polystyrene transparent multi-plate. After 20 min of reaction in the dark, changes in absorbance at 517 nm were registered for each extract. The results were expressed as inhibition percentage (INH%) (Equation (1))

(1) $$Inhibition\%=\frac{{Absorbance}_{control}-{Absorbance}_{sample}}{{Absorbance}_{control}}$$

### 4.3. Total Phenolic Content (TPC) and Total Flavonoid Content (TFC)

For each extract, the total phenolic content (TPC) was determined using the Folin–Ciocalteu method. The experiment was performed in a microplate reader (Thermo Scientific Multiskan GO, MA, United States) [63] with a 96-well polystyrene transparent multiplate. The temperature was set at 40 °C. with a 96-well polystyrene transparent multiplate. The temperature was set at 40 °C. Each extract was dissolved and diluted with methanol to 5 mg mL^−1^. Then, 30 µL of each sample was mixed with 30 µL of Folin–Ciocalteu reagent (Merck; diluted 1:10 in water). After 2 min, 240 µL of 7.5% Na_2_CO_3_ solution was added to each well. For blanks, nano-pure water was added instead of the sample extract. Finally, the absorbance at 765 nm was measured after 20 min at 40 °C. The assay was performed in triplicate for each extract. Results were presented as milligrams of gallic acid equivalents per gram of dry extract (mg GAE g^−1^ d.w.) after linear interpolation in a gallic acid calibration curve (10–80 µg L^−1^) [64].

The total flavonoid content (TFC) of each extract was evaluated using the trichloro aluminum method (AlCl3) in a Thermo Scientific Multiskan GO with a 96-well polystyrene transparent multi-plate [65]. In each well, 30 µL of each extract was mixed with 10 µL of sodium acetate (1M) and in each well, 30 µL of each extract was mixed with 10 µL of sodium acetate (1M) and 250 µL of distilled water. The absorbance was measured after 20 min at 415 nm. Results are presented in milligrams of quercetin equivalents per gram of dry extract (mg QE g d.w.^−1^) after linear interpolation in a quercetin calibration curve (10–180 µg L^−1^). All experiments were performed in triplicate [63].

### 4.4. Chemical Analysis

#### 4.4.1. HPLC-DAD

Chromatographic analysis was performed on an Agilent Technologies 1200 HPLC system (Waldbronn, Germany) with a C18e (100 × 4.6 mm I.D.) Chromolith^®^ HighResolution column (Merck, Darmstadt, Germany) and a Spectrophotometric Detector with Diode Array. The mobile phase flow rate was set at 1 mL min^−1^ and two mobile phase components were used (A: 2% acetic acid aqueous solution; B: acetonitrile) with the following mobile phase gradient program: 0–2 min, 92–91% A; 2–4 min, 91–89.8% A; 4–6 min, 89.8–88.8% A; 6–8 min, 88.8–87.5% A; 8–10 min, 87.5–86.5% A; 10–12 min, 86.5–85.2% A; 12–14 min, 85.2–84.1% A; 14–16 min, 84.1–83.0% A; 16–18 min, 83.0–81.2% A; 18–20 min, 81.2–80.9% A; 20–23 min, 80.9–75.0% A; 23–25 min, 75.0–70.0% A; 25–28 min, 70.0–65.0% A; 28–30 min, 65.0–62.0% A [62].

A 5000 mg L^−1^ extract methanolic solution was injected in triplicate with an injection volume of 20 μL. The chromatograms were registered at 278 nm. The identification was performed by comparison of the retention time and the absorbance spectra with the respective standard.

#### 4.4.2. UPLC-MS

For the mass spectrometry assays, 1 mL of each sample was transferred to a 1.5 mL centrifuge tube and then centrifuged at 13,000× *g* for 10 min. Then, 3 μL of the supernatant was injected into an Agilent (Santa Clara, CA, USA) 1290 Infinity II UPLC system using a Kinetex C18 2.1 mm × 100 mm, 1.7 μm column. The column was kept at 40 °C while the samples were kept at 6 °C. The mobile phase comprised Buffer A: 0.1% formic acid in water and Buffer B: 50:50 acetonitrile: methanol. The gradient profile was: 0 min, 0% B, 0.2 mL min^−1^; 0.5 min, 0% B, 0.2 mL min^−1^; 0.6 min, 0% B, 0.4 mL min^−1^; 4.7 min, 0% B, 0.4 mL min^−1^; 19.7 min, 97% B, 0.4 mL min^−1^; 25.2 min, 97% B, 0.4 mL min^−1^; 25.7 min, 0% B, 0.4 mL min^−1^, 27.2 min, 0% B, 0.45 mL min^−1^; stop time: 28 min. The initial 0.5 min of each run was used for calibrating the mass axis. Then, 3 μL of the sample was injected 0.7 min after the start of the run. The column temperature was 30 °C, and the autosampler temperature was 5 °C [66].

The LC was coupled to a Bruker (Billerica, MA, USA) Impact II QTOF MS (resolution > 50,000 FSR) with an ionBooster ESI source. The ESI source parameters were: End Plate Offset: 400 V, Capillary Voltage: 1000 V, Charging Voltage: 300 V, Nebulizer Pressure: 4.1 bar, Dry Gas 3 L min^−1^, Dry Gas Temperature: 200 °C, Vaporizer Temperature 350 °C, Sheath Gas 240 L/h. The mass spectrometer was operated in positive and negative mode with Auto MS/MS with the following parameters: scan range 20 to 1000 *m*/*z*, Funnel 1 RF 200 Vpp, Funnel 2 RF, 200 Vpp, isCID 0 eV, Hexapole RF 50 Vpp, Quadrupole Ion Energy 5 eV, Quadrupole Low mass 50 *m*/*z*, Collision Energy 2 eV, Pre Pulse Storage 5 μs, Scan rate 12 Hz. Fragmentation was ramped from Collision RF 200 Vpp, Transfer Time 20 μs, Collision Energy 20 to Collision RF 700 Vpp, Transfer Time 70 μs, Collision Energy 50. The mass axis was calibrated at the beginning of every sample run. Samples were measured in a randomized order. Data were acquired in Profile and Centroid Mode using Bruker Hystar 4.1 and processed using Bruker Metaboscape 5. The presence and identification of targeted phenolic compounds in all analyzed samples was determined using the software Metaboscape 4.0 from Bruker, which enables identification based on the compound mass, fragmentation pattern and isotopic pattern. Identification was confirmed using an in-house library and based on accurate mass errors (under 5 ppm) and isotopic pattern match (scores higher than 90%) as described by Edwards-Hicks et al. [66] using the parameters summarized in Table 5 and Table 6.

Feature batch annotation was performed in three ways using MetaboScape: (i) predicting the most likely sum formula with the SmartFormula tool; (ii) annotating by matching retention time, exact mass, and isotope pattern against a list of known compounds using the AnalyteList tool.and (iii) annotating by matching exact mass and fragment patterns to spectral libraries. The selected tolerances and other settings are given in Table 6. Stereochemical descriptors (e.g., D- or L-) were removed from annotated metabolite names after export from MetaboScape because the applied methodology cannot resolve stereoisomers.

### 4.5. Biological Activity

#### 4.5.1. Microbial Strains

The bacterial strains used for microbiological tests included the reference strains *Escherichia coli* ATCC 35218, *Shigella boydii* 39, *Pseudomonas aeruginosa* ATCC 1744, *Staphylococcus aureus* ATCC 25923, *Listeria monocytogenes* ATCC BAA 751, *Bacillus cereus*, *Enterococcus faecalis* ATCC 51289, *Candida guillermondii* ATCC 6260, and *Candida tropicalis* ATCC 13603. The bacterial strains were obtained from the Microbiology Laboratory collection, Department of Medical Technology, Universidad Diego Portales, kindly provided by Pedro Cortés (Ms). The strains were grown on Mueller-Hinton Agar at 37 °C [67,68]. Antimicrobials used as inhibition controls are Levofloxacin 5 µg, Surfactam-Ampicillin 10/10 µg, Ampicillin 10 µg, Chlorhexidine 2%.

#### 4.5.2. Antimicrobial Activity

##### Agar Disk Diffusion Assay

The agar disk diffusion technique has been widely used to assay plant extracts for antimicrobial activity. In this method, 6 mm sterilized filter paper disks (Whatman^®^) were saturated with 10 μL of filter-sterilized oil plant extract. The impregnated discs were then placed onto the surface of a suitable solid agar medium, such as Mueller-Hinton BD^®^. The media was pre-inoculated with test organisms. A standard inoculum size of 1 × 108 CFU/mL of bacteria was used to inoculate the diffusion plates, which is equal to the McFarland 0.5 turbidity standard. The plates were incubated overnight at 37 °C, and the diameter of the inhibition zone around each disk (diameter of the inhibition zone plus diameter of the disk) was measured in millimeters. A Sensydisc was used as a positive control for Gram+ and Gram− strains, Sigma Aldrich (St. Louis, MO, USA) [69].

##### Microplate Assay

To determine the MICs, the microdilution method in 96-well plates was employed according to the guidelines approved by the Clinical and Laboratory Standards Institute [69]. Extract of *Tristerix corymbosus* was added at 10-fold decreasing serial concentrations into Mueller Hinton BD^®^ Broth in 96-well plates (not diluted, 1:10, 1:100, and 1:1000). To inoculate the microplates, fresh bacterial suspensions were used (equivalent to 1 × 108 bacteria·mL^−1^), and the plates were incubated as previously described [67].

Extract of *Tristerix corymbosus* was solubilized in DMSO at 2% (*v*/*v*) (does not affect bacterial growth) and added to the previously inoculated microplates, and then incubated for 24 h. Absorbance was measured at 550 nm to calculate MIC, as described in the Clinical and Laboratory Standards Institute [68] protocol. Bacterial control microplates without the extract of *Tristerix corymbosus* and without bacteria were used as controls. Antibiotics were used as sensitivity-positive controls. Data were compared with ANOVA using GraphPad Prism 5 to identify significant differences between the studied groups [67].

#### 4.5.3. Cytotoxicity

Cytotoxicity was evaluated by erythrocyte osmotic fragility (or anti-hemolytic effect). In in vitro cytotoxicity assays, the integrity of the erythrocyte cell membrane in response to a substance is a good indicator to assess the biological effects induced by a compound [70]. However, in addition to the cytotoxic effects in assays such as the hemolytic activity assay, it is also necessary to determine whether a test substance exerts a protective effect on the cell membrane after hemolysis induction [71,72].

Erythrocytes isolated from the fresh blood of healthy volunteers were used. The donors had no history of chronic non-communicable diseases, nor had they consumed anti-inflammatory or cytoprotective drugs in the last 15 days. The biochemistry and basic hematology study results of their samples were within the range of normal values. 6 mL ± 10% of venous blood was obtained in vacuum blood collection tubes using the BD Vacutainer^®^ system with powdered K2 EDTA anticoagulant. Plasma was separated by centrifugation at 2500 rpm for 15 min, and the supernatant was removed. The erythrocytes were washed 4 times in phosphate-buffered saline (PBS) (pH 7.00) and resuspended in it at a rate of 8 × 10^9^ cells mL^−1^ [73] and subjected to AE (direct and diluted 1:10; 1:100, and 1:1000) for 30 min at 37 °C. The solution was then centrifuged for 10 min at 3000 rpm, the supernatant was collected, and its absorbance was measured at 540 nm using the TECAN infinite F50 reader. All experiments were performed in quintuplicate, and a 1% SDS solution was used as a positive control and PBS buffer (pH 7.4) as a negative control [74].

#### 4.5.4. Toxicity

##### Maintenance of *Caenorhabditis elegans* Culture

The wild strain of *Caenorhabditis elegans*, N2, was used for toxicity assays. The N2 strain was maintained on agar plates with nematode growth medium (NGM) in the presence of a layer of *Escherichia coli* OP50. These plates were incubated at 20 °C for 3 days. Subsequently, gravid nematodes were collected and treated in the presence of a chlorine solution (0.45 N NaOH and 2% HOCl) to obtain eggs. To hatch the eggs and obtain synchronized adult nematodes, eggs were placed on plates with OP50 for 3 days. Finally, nematodes were collected in M9 saline solution (1.5 g KH_2_PO_4_, 3 g Na_2_HPO_4_, 2.5 g NaCl, 0.5 mL of 1M MgSO_4_, and distilled water to raise the final volume to 500 mL) [58].

##### Test Preparation

The methanolic extracts of Tristerix corymbosus were prepared at concentrations of 0.78, 1.56, 3.12, 6.25, 12.50, 25.00, and 50.00 mg·mL^−1^ and added at a final volume of 100 µL to 96-well plates. Control tests were performed with 1% DMSO and M9 saline solution. *Caenorhabditis elegans* (10 adult nematodes·well^−1^) was used in each assay. The plates were incubated at 20 °C for 24 and 48 h. The experiments were performed in triplicate and repeated twice. To determine the survival rate, all nematodes were counted at 24 and 48 h. Individuals were considered alive if they presented any motility of the tail, head, or pharynx during 5 s of observation and were considered dead otherwise. Counts were obtained to determine the mortality rate [56,57].

##### Statistical Analysis

Statistical analyses were performed using the GraphPad Prism 8 software. One-way ANOVA analyses were performed to evaluate significant differences between every extract; *p* < 0.05 was considered a statistically significant difference.

## 5. Conclusions

Methanolic and chloroform extracts of leaves, and stem of *Tristerix corymbosus* were tested for their AC, TFC, TPC, chemical composition, cytotoxicity, toxicity, and antimicrobial effects on yeast, Gram-positive, and Gram-negative bacteria. Between the two solvents, methanolic extracts showed better AC, TFC, and TPC due to the higher solubility of phenolic compounds. A total of 31 compounds were tentatively identified, most of which were flavonoids, particularly, quercetin and kaempferol glycosides.

For the biological activity, while methanolic extracts from quitral show significant biological activity, their cytotoxic potential at higher concentrations necessitates further investigation into their pharmacological safety. The chloroform extracts, by contrast, may serve as promising candidates for safer applications, particularly in formulations where erythrocyte compatibility is critical.

The major compounds hyperoside, isorhamnetin-3-O-galactoside, rhamnazin, and 3-methylquercetin stand out due to their well-documented antioxidant, antimicrobial, and cytoprotective activities. These compounds appear to play a critical role in the observed biological effects, including potent free radical scavenging, moderate antimicrobial properties, and negligible hemolytic toxicity.

These findings support the potential use of this plant extract as a natural source of multifunctional agents with therapeutic relevance. Further studies involving in vivo models and mechanistic assays are warranted to fully explore its pharmacological applicability and to establish safety profiles for future development. This work contributes to the growing evidence supporting native flora as a valuable reservoir of compounds for biomedical and biotechnological innovation.

## Figures and Tables

**Table 1:** Extraction yield (%) for all analyzed extracts.

Solvent	Leaf	Stem
Methanol	3.79	8.99
Chloroform	1.58	0.30

**Table 2:** Tentatively identified compounds in QSM and QLM extracts by UPLC-MS.

Number	Compound	Classification	RT (min)	Molecular Formula	*m*/*z* Meas	Δ *m*/*z* (ppm)	Ions	QSM	QLM
1	Gallic acid	Phenolic acid	1.33	C_7_H_6_O_5_	169.0145	0.881	[M-H]-	-	-
2	Malic acid	Short-chain acid	1.61	C_4_H_6_O_5_	133.0143	0.335	[M-H]- [M-H-H_2_O]-	+	-
3	Citric acid	Short-chain acid	1.68	C_6_H_8_O_7_	191.0201	2.19	[M-H]- [M-H-H_2_O]-	+	+
4	Epicatechin	Flavonoid	3.03	C1_5_H_14_O_6_	291.0867	1.403	[M+H]+	+	-
5	2-Isopropylmalic acid	Malic acid derivative	3.06	C_7_H_12_O_5_	175.0616	2.551	[M-H]-	-	+
6	Ellagic acid	Phenolic acid	3.21	C_14_H_6_O_8_	300.9994	1.222	[M-H]-	+	+
7	Quercetin 3-O-rhamnoside 7-O-glycoside	Flavonoid	3.54	C_27_H_30_O_16_	611.1606	−0.065	[M+H]+	-	-
8	Loliolide	Monoterpene	3.58	C_11_H_16_O_3_	197.1173	1.121	[M+H]+	-	+
9	Hyperoside (quercetin-3-O-galactoside)	Flavonoid	3.79	C_21_H_20_O_12_	465.1030	0.556	[M+H]+	++	++
10	Kaempferol 3-O-sophoroside	Flavonoid	4.02	C_27_H_30_O_16_	611.1620	2.202	[M+H]+	-	-
11	Plantaginin (kaemferol-7-O-glycoside)	Flavonoid	4.09	C_21_H_20_O_11_	449.1082	0.806	[M+H]+	+	+
12	Quercetin-4′-O-glycoside	Flavonoid	4.20	C_21_H_20_O_12_	463.0882	−0.112	[M-H]-	-	+
13	Azelaic acid	Short-chain acid	4.41	C_9_H_16_O_4_	187.0977	0.781	[M-H]-	-	-
14	Isorhamnetin (3-methyl quercetin) 3-O-glycoside	Flavonoid	4.45	C_22_H_22_O_11_	461.1091	0.247	[M-H]-	-	-
15	Peonidin-3-O-beta-galactoside	Anthocyanin	4.45	C_22_H_22_O_11_	463.1238	0.95	[M+H]+	+	+
16	Homoplantaginin (6-methoxyapigenin-7-O-glycoside)	Flavonoid	4.50	C_28_H_24_O_16_	615.1005	0.857	[M-H]-	+	+
17	Quercetin-3-O-glycoside	Flavonoid	4.61	C_21_H_20_O_12_	465.1052	2.951	[M+H]+	+	+
18	Quercetin-3-O-xyloside	Flavonoid	4.69	C_20_H_18_O_11_	435.0928	1.503	[M+H]+	-	-
19	Kaempferol-3-O-glycoside	Flavonoid	4.76	C_21_H_20_O_11_	447.0945	2.06	[M-H]- [2M-H]-	-	-
20	Kaempferol-3-O-glycoside 1	Flavonoid	4.84	C_22_H_22_O_12_	477.1033	−1.211	[M-H]- [2M-H]-	+	+
21	Isorhamnetin-3-O-galactoside	Flavonoid	4.83	C_22_H_22_O_12_	479.1190	1.137	[M+H]+	+	++
22	Rutin	Flavonoid	4.91	C_28_H_24_O_16_	615.1008	2.649	[M-H]-	-	+
23	Kaempferol-3O-glcoside 2	Flavonoid	5.03	C_21_H_20_O_11_	499.1086	3.069	[M+H]+	-	+
24	Quercetin	Flavonoid	5.39	C_15_H_10_O_7_	303.0502	0.903	[M-H]-	+	+
25	Cinnamic acid	Cinnamic acid	5.74	C_9_H_8_O_2_	131.0495	2.476	[M-H_2_O+H]+	-	-
26	Rhamnazin (2′,7-dimethoxy quercetin)	Flavonoid	5.75	C_16_H_12_O_7_	317.0662	1.915	[M+H]+	++	+
27	3-methylquercetin	Flavonoid	5.76	C_16_H_12_O_7_	315.0503	−2.273	[M-H]-, [2M-H]-	++	+
28	Kaempferol	Flavonoid	6.32	C_15_H_10_O_6_	287.0558	2.752	[M+H]+	-	-
29	Isokaempferol	Flavonoid	6.64	C_16_H_12_O_6_	299.0553	−1.581	[M-H]- [2M-H]-	-	-
30	Quercetin-3,7-dimethileter	Flavonoid	6.77	C_17_H_14_O_7_	331.0819	2.002	[M+H]+	+	-
31	Avobenzone	Curcumin derivative	12.53	C_20_H_22_O_3_	311.1643	0.349	[M+H]+	+	n.d.

++ area > 10^+5^, + area > 10^+4^, - area > 10^+2^, n.d. not detected.

**Table 3:** Antimicrobial activity of all extracts tested against 7 bacterial strains and 2 yeast strains expressed as inhibition diameter (mm).

Microorganism	Negative Control	Positive Control	QLM	QSM	QFM	QLC	QSC	QFC
*E. coli*	-	34.0 ^1^	-	-	-	-	-	-
*S. boydii*	-	29.0 ^2^	13.5	12.0	15.0	-	-	-
*P. Aeruginosa*	-	25.0 ^1^	-	12.0	9.4	-	-	-
*S. Aerus* ATCC 25923	-	55.0 ^3^	11.5	-	-	-	-	-
*L. monocytogenes* ATCC BAA751	-	36.0 ^3^	17.5	-	-	15.0	26.0	-
*B. cereus*	-	15.0 ^3^	10.0	10.0	7.0	8.0	-	7.0
*E. faecalis*	-	24.0 ^2^	-	-	-	-	-	-
*C. guillermondii*	-	32.0 ^4^	-	-	-	-	-	-
*C. tropicalis*	-	21.0 ^4^	-	-	-	-	-	-

^1^ Levofloxacin, ^2^ Surfactam-Ampicillin, ^3^ Ampicillin, ^4^ Chlorhexidine.

**Table 4:** MICs (μg·μL^−1^) of QLM, QSM, QLC, and QSC against bacteria (clinical isolates and ATCC).

Microbial Strain	QLM	QSM	QLC	QSC
*S. boydii*	34.0	332.5	67.4	67.4
*P. Aeruginosa*	32.5	67.4	67.4	67.4
*S. Aerus* ATCC 25923	34.0	67.4	67.4	68.0
*L. monocytogenes* ATCC BAA751	32.5	68.0	67.4	32.5
*B. cereus*	68.0	68.0	67.4	68.0

The ANOVA analysis, including the Tukey test, did not show significant differences between QLM, QSM, QLC and QSC. The whole experiment was performed in triplicate.

**Table 5:** Data processing parameters. Includes batch feature annotation in Metaboscape 5.

**Filter Parameters**	
Minimum # Features for Extraction	1
Presence of features in minimum # of analyses	Processing Parameters3
**T-ReX 3D**	
Intensity threshold	4000 (polar metabolites)
Minimum Peak Length	12
Enable Recursive Feature Extraction	True
Minimum Peak Length (recursive)	7
Perform MS/MS import	True
MS/MS import method	Average
**Ion Deconvolution Parameters**	
EIC correlation	0.8
Primary ion (negative mode)	[M-H]-
Primary ion (positive mode)	[M+H]+
Seed ions (negative mode)	[M+Cl]-
Seed ions (positive mode)	[M+Na]+, [M+K]+, [M+NH_4_]+
Common ions (negative mode)	[M-H-H_2_O]-, [M+COOH]-
Common ions (positive mode)	[M-H-H_2_O]+
**Mass Calibration Parameters**	
Lock Mass Calibration	False
Mass Recalibration	True, calibration segment 0.1-0.4 min
**Expert settings**	
FerraWorkflow.chargeMax	1 (only for polar metabolites)

**Table 6:** Settings and parameters for feature annotation in Metaboscape.

**Smart Formula Parameters**	
*m*/*z* tolerance	1 mDa (narrow), 3 mDa (wide)
mSigma	15 (narrow), 50 (wide)
Elements	CHNOPS
Upper formula	S1
Element ratio filters	Common
Electron configuration	Both
**Analyte List Parameters**	
*m*/*z* tolerance	1 mDa (narrow), 3 mDa (wide)
Retention time tolerance	0.2 min (narrow), 0.4 min (wide)
mSigma	15 (narrow), 50 (wide)
**Spectral Library Parameters**	
Libraries (polar metabolites)	In house library, Bruker MetaboBASE 3.0, GNPS export (downloaded July 2020)
*m*/*z* tolerance	1 mDa (narrow), 3 mDa (wide)
mSigma	20 (narrow), 200 (wide)
MS/MS score	900 (narrow), 700 (wide)

## Data Availability

The original contributions presented in this study are included in the article. Further inquiries can be directed to the corresponding author.

## Abbreviations

The following abbreviations are used in this manuscript:

AC	Antioxidant Capacity
AUC	Area under the curve
DPPH	2,2-Diphenyl-1-picrylhydrazy
LC	Liquid Chromatography
MIC	Minimum Inhibitory Concentration
ORAC	Oxygen Radical Absorbance Capacity
QE	Quercetin Equivalent
QLC	Quitral Leaves Chloroform
QLM	Quitral Leaves Methanol
QSC	Quitral Stem Chloroform
QSM	Quitral Stem Methanol
TE	Trolox Equivalent
TFC	Total flavonoid Content
TPC	Total Phenolic Content
